# New aesthetic in-house 3D-printed brackets: proof of concept and fundamental mechanical properties

**DOI:** 10.1186/s40510-022-00400-z

**Published:** 2022-02-21

**Authors:** Spyridon N. Papageorgiou, Georgios Polychronis, Nearchos Panayi, Spiros Zinelis, Theodore Eliades

**Affiliations:** 1 Clinic of Orthodontics and Pediatric Dentistry, Center of Dental Medicine, University of Zurich, Plattenstrasse 11, 8032 Zurich, Switzerland; 2 Department of Biomaterials, School of Dentistry, National and Kapodistrian University of Athens, Athens, Greece; 3 Department of Dentistry, European University Cyprus, Nicosia, Cyprus

**Keywords:** Orthodontic brackets, Resins, 3D printing, Mechanical properties, Instrumented indentation testing

## Abstract

**Objectives:**

Three-dimensional (3D) printing technology is an emerging manufacturing process for many orthodontic appliances, and the aim of this study was to evaluate the mechanical properties of resin-based materials as alternatives for the in-house preparation of orthodontic brackets.

**Material and Methods:**

Two types of 3D printed resins used for temporary (T) and permanent (P) crown fabrication were included in this study. Ten blocks from each resin were manufactured by a 3D printer and, after embedding them in acrylic resin, the samples were subjected to metallographic grinding and polishing, followed by instrumented indentation testing (IIT). Martens hardness (HM), indentation modulus (*E*_IT_), and elastic index (*η*_IT_) were determined with a Vickers indenter recording force-indentation depth curves from each specimen. After calculating descriptive statistics, differences between material types were investigated with Wilcoxon rank sum test accounting for clustering of measurements within specimens at alpha = 5%.

**Results:**

No statistically significant differences in the mechanical properties of the two tested materials were seen: HM: median 279 N/mm^2^ (interquartile range [IQR] 275–287 N/mm^2^) for T and median 279 N/mm^2^ (IQR 270–285 N/mm^2^) for P (*P* value = 0.63); *E*_IT_: median 5548 MPa (IQR 5425–5834 MPa) for T and median 5644 (IQR 5420–5850 MPa) for P (*P* value = 0.84); *η*_IT_: median 47.1% (46.0–47.7%) for T and median 46.0% (IQR 45.4–47.8%) for P (*P* value = 0.24).

**Conclusions:**

Under the limitations of this study, it may be concluded that the mechanical properties of the two 3D printed resins tested are equal, and thus, no differences in their clinical performance are expected.

## Introduction

Aesthetic brackets present an alternative to the classic metallic fixed appliances, which is highly desirable among adults and even some adolescent patients. However, their optical superiority over the metallic brackets does not necessarily directly translate to their mechanical properties as well. Ceramic brackets, for instance, have increased brittleness that can result to wing fractures [1, 2], impacts of the archwire surface on their surface [3], as well as wear of antagonist teeth [4]. Furthermore, their inability to plastically deform is associated with difficulties during the debonding process and with increased risk of damaging tooth enamel [5]. On the other hand, plastic brackets are soft and compliant, undergo intra-oral plasticization, and offer minimal resistance to deformation [6–8]. As a result, these lead to reduced material longevity, impaired tooth movement accuracy, and troublesome torque implementation [7, 9, 10], which result in a questionable efficacy of the orthodontic therapy. Although controversial, both types seem to impede sliding mechanics due to friction enhancement [11–13]. Taking into account these findings, it becomes clear that material selection is of paramount importance for the clinical performance of aesthetic brackets.

**Fig. 1:**
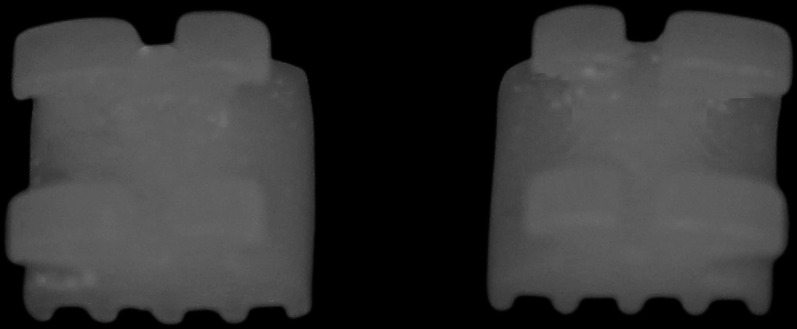
Prototype resin bracket manufactured by 3D printing technology

Apart from the raw material per se, the manufacturing method can also influence considerably the material properties, since it can lead to the presence of material imperfections or defects, generation of stress concentration areas, or partial polymerization—all of which may have detrimental consequences on the end product’s strength, modulus of elasticity, and hardness. Additive technology in particular has become a highly popular solution for the construction of orthodontic appliances as it reduces costs, increases efficiency, and offers many customization possibilities [14, 15]. The technique is based on 3D models stored in a computer and utilizes a printer for fabricating the appliance in an incremental fashion layer by layer. Some first attempts to produce ceramic brackets [16] or resin-based ones have been reported [14, 17], but in general the selection of an appropriate new material for printing aesthetic brackets that bypass the inherent problems of commercially available ones still remains a challenge. Figure 1 demonstrates a pair prototype bracket manufactured from resin by 3D-printed technology.

However, to the best of our knowledge the mechanical properties of the resin-based materials that can be used by 3D printing technology for the production of orthodontic brackets are still unknown, and thus, the aim of this study is to investigate these properties of two available resin based materials for this purpose. The null hypothesis was that there is no significant difference between the properties of these two materials.

## Materials and methods

Two types of resins used for the 3D printing of temporary (T) (temporary CB resin, Formlabs, Somerville, MA, USA) or permanent (P) (permanent crown resin, Formlabs, Somerville, MA, USA) crown fabrication comprised our sample. Ten blocks with dimensions 10 mm × 10 mm × 1.5 mm from each type were designed using the computer-aided design software Meshmixer (Autodesk, San Rafael, California, USA). The blocks were exported from the software and virtually positioned on the 3D printer’s (Formlabs 3B, Somerville, Massachusetts, USA) platform to be manufactured by it. Printing time was approximately 20 min for both samples. After printing, the samples were immersed and washed in the Formwash machine (Formlabs, Somerville, MA) using isopropyl alcohol for 15 min. The samples were then cured in the Cure M curing unit (Graphy, Seoul, Korea) for 20 min on each side.

The specimens were embedded in acrylic resin (Verso Cit-2, Struers, Ballerup, Denmark), ground up to 4000 grit-size SiC abrasive grinding papers under water cooling, polished with a water-based diamond suspension (NapR1 DiaPro, Struers, Ballerup, Denmark) of 1 µm particle size in a grinding/polishing machine (Dap-V, Struers, Ballerup, Denmark), and subjected to instrumented indentation testing (IIT). Martens hardness (HM), indentation modulus (E_IT_), and elastic index (η_IT_) were assessed. Testing was conducted in a universal hardness testing machine (ZHU0.2/Z2.5, Zwick Roell, Ulm, Germany) with a Vickers indenter at ambient temperature. The HM, *E*_IT_, and *η*_IT_ were acquired from force–indentation depth curves applying a maximum load of 4.9 N for a 2 s contact time. Six measurements from each block were taken into consideration to determine the specimen’s mechanical properties, which were calculated according to formulas provided by the international standard ISO14577-1 [18].

After checking for normality through visual inspection and formally with the Shapiro–Wilk test, descriptive statistics were calculated including medians and interquartile ranges (IQR). Differences between T and P crown materials were checked through Wilcoxon rank sum test accounting for clustering of measurements within each specimen at alpha = 5%. All analyses were run in R Software version 4.0.3 (R Foundation for Statistical Computing, Vienna, Austria) with an open dataset [19].

## Results

**Fig. 2:**
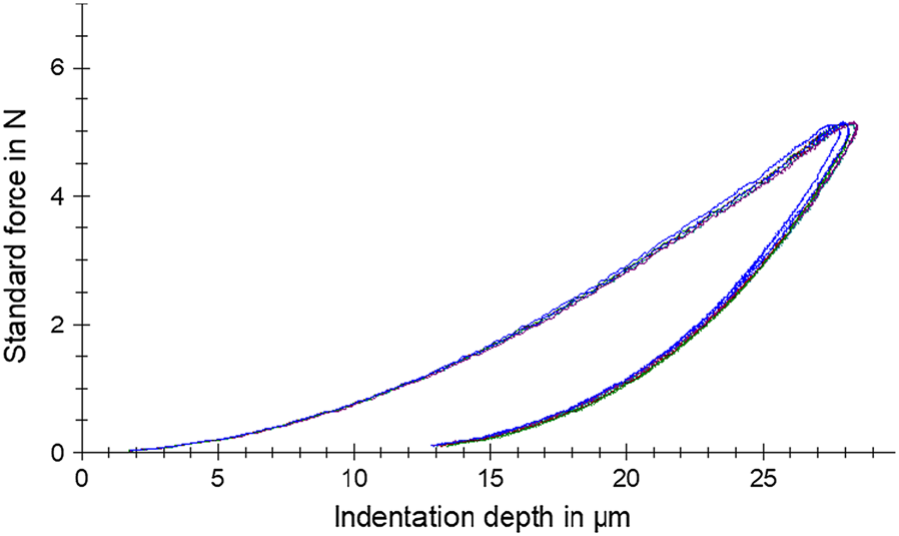
Representative force-indentation depth curves from both groups tested

Figure 2 shows a selection of force-indentation depth curves from both groups. All curves are similar in shape and position without any obvious differences. The results of all mechanical properties including their statistical analysis are presented in Table 1. No statistically significant differences between temporary and permanent resins were found for HM (medians of 279 and 279 N/mm^2^, respectively; *P* = 0.63), *E*_IT_ (medians of 5548 and 5644 MPa, respectively; *P* = 0.84), or *η*_IT_ (medians of 47.1 and 46.0%, respectively; *P* = 0.24).

**Table 1:** Results of the instrumented indentation testing

Property	Temporary Mean (IQR)	Permanent Mean (IQR)	P value
HM (N/mm^2^)	279 (275, 287)	279 (270, 285)	0.63
E_IT_ (MPa)	5548 (5425, 5834)	5644 (5420, 5850)	0.84
η_IT_ (%)	47.1 (46.0, 47.7)	46.0 (45.4, 47.8)	0.24

IQR, interquartile range

## Discussion

Since no significant differences were observed between groups, the null hypothesis cannot be rejected, and therefore, the scenario of the two materials having similar mechanical properties is compatible with the data. The materials under investigation underwent IIT testing, which provided crucial information about fundamental mechanical properties such as their hardness, modulus of elasticity, and brittleness (elastic index) [18]. The method itself is highly versatile as there is no need for rectangular or cylindrical specimens and circumvents the classic measurement of residual indentation and the related limitations, providing fully automated and highly reliable results [20].

The 3D-printed resins showed mechanical characteristics substantially superior than the commercially available plastic brackets. The theoretical Vickers Hardness was also calculated for the tested material (35 HV) just for comparison purposes with previously published data and found almost 2 times higher compared to HV reported for plastic brackets (19.6–16.9 HV) [21] In particular, hardness which is defined as the resistance to indentation and concomitantly is a measure of wear resistance was found to be 270 N/mm^2^ which approaches the hardness of stainless steel bracket wings (360 N/mm^2^) [22]. However, as with the case of stainless steel brackets a concern may arise when the hardness of archwires is considered; this may range from 1500 to 2600 MH, depending on the alloy used with the nickel-titanium alloys being toward the lower side and the stainless steel ones being on the high side [22, 23]. As a rule, a mismatch in hardness is not desirable since it promotes wear across the path of the archwire into the slot. The harder material will leave an imprint in the softer and in most cases that is the stainless steel or nickel-titanium wire leaving an imprint in the plastic brackets. The opposite is happening on the other side in ceramic brackets. The clinical significance of this finding about hardness may pertain to the fact that low hardness wing may impede the movement of bracket along the buccal wire segment in case of sliding mechanics or complicate the transfer of torque from an activated archwire to the bracket, as well as may preclude full engagement of the wire to the slot wall and possible plastic deformation of the wing [24]. An increased hardness is necessary to facilitate surface integrity and preclude binding of the wire onto the bracket slot walls. This problem had been encountered with conventional plastic brackets as well as metal injection molding (MIM) metallic brackets. The latter are cast appliances, and as a result, the traditional concept of low hardness low modulus bracket base to facilitate uneventful debonding and high hardness high modulus wings to withstand the forces and moments developed during mechanotherapy could not be applied [25].

As far as modulus of elasticity is concerned (the higher the modulus, the higher the stiffness of a structure), the tested resinous materials (5.5–5.6 GPa; Table 1) demonstrate much lower modulus of elasticity compared to alumina brackets (138–141 GPa) [26] and metallic brackets made of stainless steel alloys (62–83 GPa) [22]. This property has various implications on the understanding of several phenomena encountered during clinical practice. As high moduli of elasticity imply high resistance to deformation, they are desirable for areas where no deflection is required. These include the brackets slot walls and wings, which should not be compliant to allow for efficient transmission of the loads applied by an activated archwire to the tooth. To this end, ceramic brackets show higher stiffness as a result of the arrangement of atoms and bonding inside their structure, and for this reason, they present better performance when it comes to transmission of loads, whereas plastic brackets apart from their lower stiffness, which in most cases make them unsuitable for this task, show also several other disadvantages such as potential release of bisphenol A. Even though a high modulus of elasticity is preferred for some components of the bracket, for other parts, such as the bracket base, this is an undesirable feature [24]. This is due to the difficulty in squeezing a stiff base at debonding, which necessitates the application of increased forces; this effect coupled with sensitive and / or sore teeth increases the discomfort/pain during debonding and iatrogenic trauma on tooth enamel [26, 27]. Future developments on this field could include the heavy filling of resin used for the 3D printing of brackets which would improve its modulus of elasticity.

Elastic index is indicative of a material’s brittleness [28], and the higher the elastic index the higher the brittleness, and from this standpoint a lower elastic index is desirable for orthodontic appliances. Besides, the clinical failure of ceramic alumina brackets during therapy or debonding [29, 30] has been associated with their brittle nature, which is reflected on the absence of plastic deformation and low fracture toughness [26]. The tested materials presented intermediate values of elastic index (46–47%), which are somewhere between the values of alumina (55–62%) [26] and the values of stainless steel brackets (15–22%) [26]. This means that they are less susceptible to chipping or fracture compared to alumina brackets, even though their behavior still remains inferior compared to metallic ones at this perspective.

Apart from the mechanical properties considered in the foregoing discussion, in-house, 3D-printed plastic brackets offer significant advantages with respect to appliance design and features. The deviation from the concept of a standard-sized and prescription bracket offers the opportunity to arrange these two characteristics per malocclusion variation. Therefore, the width of the bracket may be increased in cases of large rotations or inclinations to accommodate improved rotational and tip control; the torque prescription can be altered in scenarios where larger maxillary anterior torque is needed to maintain torque or regain it during retraction; and the thickness of the bracket can also be modulated to account for posterior teeth, most often maxillary second premolars which tend to be smaller in the buccolingual dimension and therefore in some extraction cases contribute to the formation of black corridors in the buccal segment after extraction [31].

Moreover, the customized 3D printing offers the ability to individually arrange and assess the prescription values which have been shown to vary extensively in manufactured bracket slots [32].

## Conclusions

Under the limitations of this research, the 3D printed resins tested exhibited mechanical properties superior than contemporary plastic brackets, whereas they offer the unique feature of customized size, shape, and prescription according to specific characteristics of the malocclusion treated.

## Data Availability

All data generated or analyzed during this study are included in this published article or its supplements, while its dataset is openly provided through Zenodo (http://doi.org/10.5281/zenodo.5574399).

## Abbreviations

3D: Three-dimensional
E_IT_: Indentation modulus
HM: Martens hardness
IIT: Instrumented indentation testing
IQR: Interquartile range
P: Permanent
T: Temporary
η_IT_: Elastic index
